# A High-Sensitivity Method for Detection and Measurement of HMGB1 Protein Concentration by High-Affinity Binding to DNA Hemicatenanes

**DOI:** 10.1371/journal.pone.0002855

**Published:** 2008-08-06

**Authors:** Claire Gaillard, Chloé Borde, Joël Gozlan, Vincent Maréchal, François Strauss

**Affiliations:** Centre de Recherche des Cordeliers, Université Pierre et Marie Curie, Université Paris Descartes, INSERM, Paris, France; University of Hong Kong, China

## Abstract

**Background:**

Protein HMGB1, an abundant nuclear non-histone protein that interacts with DNA and has an architectural function in chromatin, was strikingly shown some years ago to also possess an extracellular function as an alarmin and a mediator of inflammation. This extracellular function has since been actively studied, both from a fundamental point of view and in relation to the involvement of HMGB1 in inflammatory diseases. A prerequisite for such studies is the ability to detect HMGB1 in blood or other biological fluids and to accurately measure its concentration.

**Methodology/Principal Findings:**

In addition to classical techniques (western blot, ELISA) that make use of specific anti-HMGB1 antibodies, we present here a new, extremely sensitive technique that is based on the fact that hemicatenated DNA loops (hcDNA) bind HMGB1 with extremely high affinity, higher than the affinity of specific antibodies, similar in that respect to DNA aptamers. DNA-protein complexes formed between HMGB1 and radiolabeled hcDNA are analyzed by electrophoresis on nondenaturing polyacrylamide gels using the band-shift assay method. In addition, using a simple and fast protocol to purify HMGB1 on the basis of its solubility in perchloric acid allowed us to increase the sensitivity by suppressing any nonspecific background. The technique can reliably detect HMGB1 at a concentration of 1 pg per microliter in complex fluids such as serum, and at much lower concentrations in less complex samples. It compares favorably with ELISA in terms of sensitivity and background, and is less prone to interference from masking proteins in serum.

**Conclusion:**

The new technique, which illustrates the potential of DNA nanoobjects and aptamers to form high-affinity complexes with selected proteins, should provide a valuable tool to further investigate the extracellular functions of HMGB1 and its involvement in inflammatory pathologies.

## Introduction

Protein HMGB1 (High Mobility Group B1) was initially discovered as one of the most abundant nuclear non-histone proteins in eukaryotic cells. After its discovery [1] it was mostly studied for its interactions with DNA and its function in the nucleus as a constituent of chromatin [2], and as the prototype of the HMG-box family of proteins, many of which are implicated in the control of differentiation or development [3]. For many years the extranuclear functions of HMGB1 were little studied (with the notable exception of studies on amphoterin, the other name of HMGB1, by Rauvala and coworkers [4]), until Wang et al. [5] strikingly showed that, in addition to its nuclear functions, HMGB1 was involved in sepsis as a late mediator of endotoxin lethality in mice. HMGB1 has since been implicated in many other pathologies including arthritis and cancer, and many works have studied the function of extracellular HMGB1 in infectious and inflammatory disorders, its interest as a potential therapeutic target, and its role as a messenger («alarmin») when released from the nuclei of necrotic cells (for recent reviews see e.g. [6]–[10]).

**Figure 1:**
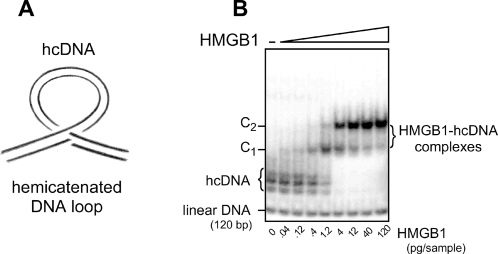
(A) Schematic representation of a hemicatenated DNA loop (hcDNA). A double-stranded DNA fragment is folded into a loop maintained at its base by a hemicatenane, i.e. the junction of two double-stranded DNA molecules in which one of the strands of one duplex passes between the two strands of the other duplex, and reciprocally. (B) Gel electrophoresis of hcDNA and its complexes with HMGB1. A 120 bp DNA fragment was ^32^P end-labeled and used to prepare hcDNA, which was analyzed by electrophoresis on a 4% polyacrylamide gel. hcDNA (left lane) migrates as several bands as a function of the size and of the location of the loop along the structure. Addition of the indicated amounts of pure HMGB1 results in the formation of DNA-protein complexes that migrate as two retarded bands, C1 and C2, corresponding to the binding of one or two HMGB1 molecules per hemicatenane, respectively. For the low protein concentrations the amount of shifted hcDNA is proportional to the protein amount, with a lower detection limit of 0.04 pg HMGB1 [21]. At high protein concentrations, when all the hcDNA probe is complexed with HMGB1, the amount of hcDNA becomes limiting and further addition of HMGB1 does not modify the band pattern.

Experimentally, most of these studies require the ability to detect HMGB1 and measure its concentration in cell culture medium or in biological samples, most frequently blood plasma or serum. Initially the western blotting technique was most widely used [5], [11], [12], while more recently ELISA have become available [13]–[15]. In both techniques the detection of HMGB1 rests on the high specificity and affinity of antibodies directed against HMGB1. However antibodies are not the only macromolecules with very high affinity for HMGB1, as this protein also presents a strong affinity for specific alternative conformations of DNA. Indeed, since the first observation of HMGB1 binding to DNA cruciforms [16], several DNA substrates with higher and higher affinities have been identified, culminating with DNA minicircles of less than 100 bp [17]–[19], (dissociation constant *K*_d_ ∼100 pM), and with hemicatenated DNA loops (hcDNA) [20], which presently constitute the HMGB1 substrate with the highest affinity known, with a dissociation constant *K*_d_<0.15 pM [21], [22]. The structure of hemicatenated DNA loops is schematically represented in Figure 1A; they consist in a DNA loop maintained at its base by a DNA hemicatenane, i.e. the junction of two double-stranded DNA molecules in which one of the strands of one molecule passes between the two strands of the other, and reciprocally, forming a DNA knot [20] that could be visualized by atomic force microscopy (AFM) [23]. Figure 1B shows that this structure, formed here with a 120 bp DNA fragment and analyzed on a non-denaturing polyacrylamide gel, migrates slightly more slowly than linear DNA and associates with HMGB1 to form very stable complexes that can easily be detected on polyacrylamide gels due to their reduced mobility as compared to the unbound hemicatenated probes.

In addition to studying the role of hemicatenanes and their interactions with HMGB1 in genome structure and function [24], we also considered taking advantage of the extremely high affinity of hcDNA for HMGB1, superior to the average affinity of specific antibodies [25], to detect HMGB1 and to measure its concentration in biological fluids. Here we show that HMGB1 can be detected at extremely low concentrations in all kinds of samples, especially in serum. The property of HMGB1 to be soluble in acid allowed us to design a simple and rapid protocol to purify it from most other serum proteins, reducing the nonspecific background and greatly increasing the sensitivity of the technique. Finally we discuss the characteristics of this new technique and its advantages relative to techniques such as ELISA that are based on the binding of HMGB1 to specific antibodies.

## Materials and Methods

### HMGB1

Protein HMGB1 of mammalian origin, either from rat liver nuclei or from cultured cells (human HeLa or monkey CV1 cell lines), was purified by chromatography as described [26]. Alternatively HMGB1 was expressed in *E. coli* as a fusion protein with a N-terminal (His)_6_ tag, using a plasmid construct made by inserting the rat HMGB1 gene into expression vector pET15b [22]. The His-tagged protein was purified by affinity chromatography on Ni-NTA, followed by ion exchange chromatography on a mono P column [27]. The concentration of the purified proteins was determined spectrophotometrically and further confirmed by electrophoresis on SDS-polyacrylamide gels, coomassie blue staining, and comparison with samples containing known amounts of a control protein [22]. Measurement of HMGB1 concentration by enzyme-linked immunosorbent assay (ELISA) was performed using the HMGB1 ELISA kit from Shino-Test Corporation, Japan, according to the manufacturer's protocol.

### Preparation of hcDNA

hcDNA was prepared from a DNA fragment containing a tract of poly(CA)·poly(TG), essentially as described previously [20]. In brief, the fragment is heat-denatured and allowed to renature in the presence of HMGB1; shifted reassociation of the repetitive sequences leads to DNA folding into a hemicatenated loop.

In practice, the DNA fragment used to prepare hcDNA originated from plasmid pE10 (accession X96980), a pUC19 derivative containing a 62 bp tract of the repetitive DNA sequence poly(CA)·poly(TG). A 120 bp fragment containing the repetitive tract was prepared by digestion of this plasmid with EcoRI and ClaI, polyacrylamide gel purified, dephosphorylated, and end-labeled with ^32^P using polynucleotide kinase and [γ-^32^P]ATP (6 000 Ci/mmol≈220 000 Bq/pmol), following standard protocols.

For the shifted strand reassociation leading to hcDNA formation, two 1.5 mL tubes are prepared, the first tube containing ∼200 ng of labeled DNA fragment in 50 µL TE (10 mM Tris-HCl, 1 mM EDTA, pH 7.5), the second tube containing 20 µL of 10× buffer (500 mM NaCl, 150 mM Tris-HCl pH 7.5, 10 mM DTT, 1 mM EDTA, 1% Triton X-100), 100 ng bacteriophage lambda DNA, 1 µg HMGB1, plus TE buffer to a final volume of 150 µL. The DNA fragment present in the first tube is denatured by a 3 min incubation in a water bath at 100°, then transferred and mixed as quickly as possible to the content of the second tube and incubated 30 min at 37°.

The hcDNA-HMGB1 complex formed, which may contain up to 25–30% of the total labeled DNA, is purified by electrophoresis on a 4% polyacrylamide gel (see below) and electroelution. HMGB1 is removed by a 2 min extraction with chloroform at room temperature in the presence of 1 M NaCl and 1% SDS, followed by 5 min centrifugation at 13 000×g at 4°. The aqueous phase is recovered and hcDNA precipitated with 2.5 vol. ethanol in the presence of linear polyacrylamide carrier [28], redissolved in TE +50 mM NaCl, and stored at 4° in low-binding polypropylene tubes.

#### Comments

The presence of HMGB1 during the formation process of hcDNA is required to increase DNA flexibility and facilitate the formation of a loop. Whether HMGB1 originates from mammalian cells or is a His-tagged recombinant protein makes no significant difference.

The denatured DNA must be transferred as quickly as possible to the solution containing HMGB1, as premature reassociation of DNA strands would reduce the yield of hcDNA. The specific activity of the probe does not depend on this yield, however, but remains equal to the specific activity of the labeled DNA fragment used to prepare hcDNA.

The presence of competitor DNA is required to avoid smearing on the preparative gel due to non-specific binding of HMGB1 to labeled DNA. This is particularly important when His-tagged HMGB1 is used, as the affinity of the tagged protein for linear DNA is somewhat higher than the affinity of HMGB1 from mammalian origin (C.G. and F.S., unpublished data).

The use of DNA from bacteriophage lambda as a competitor, or of any other DNA with a high and well-defined molecular weight, avoids any contamination of hcDNA with short fragments of competitor DNA. However such a contamination would have no visible effect on the band shift assay, it is therefore possible to use any common DNA such as salmon sperm or *E. coli* DNA as competitor.

### hcDNA-HMGB1 interactions

Incubations are performed in low-binding polypropylene tubes containing labeled hcDNA (∼5000 cpm) in 25 µL of 50 mM NaCl, 25 mM Tris-HCl pH 7.5, 1 mM DTT, 1 mM EDTA, 0.1% Triton X-100, 100 µg/mL BSA, 2% glycerol, plus unlabeled competitor DNA. Double-stranded competitor was full-length DNA from bacteriophage lambda, but any double-stranded DNA would be equally efficient. Single-stranded competitor was heat-denatured DNA from *E. coli*; here again any DNA can be used provided it is of sufficient complexity to avoid its fast renaturation during incubation. Unless otherwise noted, the amounts of competitor DNA used are the following. With pure HMGB1: no competitor. With samples containing low concentrations of proteins, such as cell-culture medium: 100 ng double strand +10 ng single strand DNA per sample. With serum (or plasma) not treated with perchloric acid: 2 µg double strand +1 µg single strand. With serum after perchloric acid precipitation: 100 ng double strand, no single strand.

To hcDNA in the above solution, a given volume, 1 to 2 µL, of sample to be assayed is added and gently mixed. After 20 min of incubation at 37° the samples are loaded onto a polyacrylamide gel. For quantitative measurement of HMGB1 concentration, calibration curves are obtained using serial dilutions of a known amount of HMGB1 in 50 mM NaCl, 25 mM Tris-HCl pH 7.5, 1 mM DTT, 0.1 mM EDTA, 0.1% Triton-X100, 100 µg/mL BSA, 15% glycerol. Triton-X100 and BSA are both required for dilution of HMGB1, even when working with low-binding polypropylene tubes, to avoid any loss of HMGB1 on tube walls and to ensure that the HMGB1 concentrations of the serial dilutions are as accurate as possible. Dilute HMGB1 solutions are stored frozen at −80°, and can be thawed and frozen several times with no significant loss of hcDNA-binding activity. The calibration curves were found to depend on two parameters only: the amount of hcDNA probe per sample is obviously the most important parameter; second, as HMGB1 presents a weak but detectable affinity for single-stranded DNA, a decrease of the signal is observed in the presence of high concentrations of single-stranded competitor.

The anti-HMGB1 antibody used to supershift the HMGB1-hcDNA complexes was affinity-purified rabbit anti-HMG1 polyclonal antibody from BD Pharmingen (now discontinued).

### Band-shift assay

In initial experiments [20], [22], the complexes of HMGB1 with hcDNA were analyzed by electrophoresis on 4% polyacrylamide gels in a low-ionic strength buffer (6.7 mM Tris, 3.3 mM Na Acetate, 1 mM EDTA, pH 7.8), at 4°, with buffer recirculation, a gel system often used to stabilize DNA-protein interactions. Later we observed that the hcDNA-HMGB1 complexes were so stable that they could be analyzed with identical results in 4% polyacrylamide gels in 0.5× TBE buffer at room temperature without buffer recirculation, a much more convenient gel system. Consequently complexes are now routinely analyzed at room temperature on 4% polyacrylamide gels (acrylamide∶bis-acrylamide 29∶1) in 0.5× TBE buffer at a voltage of ∼8 V/cm, with a 10 min prerun and 3 hr of migration for a 15 cm gel. After electrophoresis the gels are dried on a sheet of Whatman 3 MM paper and exposed either on autoradiographic film or on a phosphorimager screen for quantification of the radioactivity in the bands.

Calibration of the technique is performed with a series of samples containing known amounts of pure HMGB1, assayed in parallel under strictly identical experimental conditions. After exposure of the gel to autoradiographic film or phosphorimager screen, the intensities of the bands are measured with ImageJ or ImageQuant software, respectively. A calibration curve is drawn by plotting the percentage of shifted material (bands C1+C2) as a function of the amount of HMGB1 on a semi-logarithmic scale. A second calibration curve, which gives useful information for high-concentration samples when 100% of hcDNA is shifted, is drawn by plotting the ratio of intensities of peaks C2 and C1 as a function of HMGB1 amount.

### Viral infections

Human epithelial cells of genital origin (HEC-1) were grown in RPMI medium supplemented with 5% fetal calf serum (or 1% serum during viral infection), at 37°C in the presence of 5% CO_2_ until sub-confluence. Infections were performed in 6-well plates with a clinical isolate of Herpes Simplex Virus type 2 at various multiplicities of infection. Culture medium was collected at the indicated times after infection and clarified by centrifugation (13 000×g, 10 min, 4°C) before analysis.

### Perchloric acid precipitation

The majority of serum proteins other than HMGB1 can be removed by precipitation in the presence of 3% perchloric acid (PCA) as follows.

A stock solution of 13.7% PCA is prepared by mixing 1.26 vol. of 70% PCA with 8.69 vol. H_2_O. These values, especially the value of 13.7% for the PCA stock, have been derived from the tables of perchloric acid density as a function of concentration [29] so as to yield a final PCA concentration of 3% upon five-fold dilution. The PCA stock solution is stored in an air-tight brown-glass bottle at room temperature.

To 1 vol. of serum (or other sample to be analyzed) on ice, ¼ vol. PCA at 13.7% is added and mixed. The 3% PCA concentration results in the instant formation of a very abundant precipitate. The sample is immediately centrifuged for 5 min. at 13 000×g at 4° in a benchtop centrifuge. A known volume of the supernatant, which contains HMGB1, is collected and neutralized by addition of 1/5 its volume of 1.5 M NaOH.

As the precipitation-neutralization process results in a 50% volume increase due to the addition of reagents, 1.5 µL of the neutralized supernatant is assayed to measure HMGB1 concentration in 1 µL of serum.

## Results

The band-shift assay is an extremely sensitive technique that is particularly suited to study high-affinity DNA-protein interactions such as HMGB1 binding to hcDNA [21], [22]. The gel shown in Figure 1B illustrates the results obtained with a hcDNA probe prepared from a DNA fragment ^32^P-end-labeled to high specific activity. With serial dilutions of a highly purified preparation of HMGB1 from mammalian cells, one can detect 0.04 pg of HMGB1, an amount that gives a weak but clearly visible signal. With 1 pg of protein, more than half of the probe is bound by HMGB1. Upon further increasing the protein concentration the signal reaches a plateau when the amount of substrate becomes limiting, as one hcDNA molecule cannot bind more than two HMGB1 molecules. Note that in this experiment the labeled double-stranded DNA that is present with the probe is not bound at all by HMGB1.

**Figure 2:**
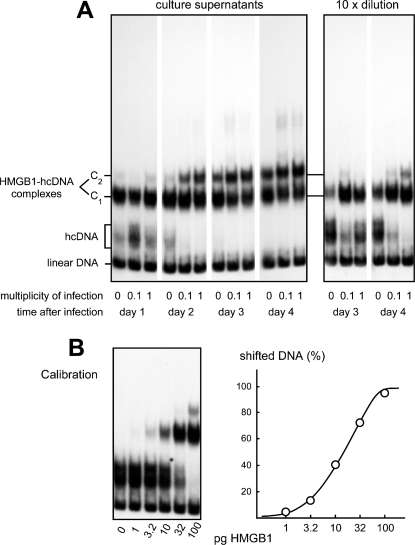
HMGB1 protein assay in culture medium of epithelial human cells infected by herpes simplex virus type 2. (A) Cell culture medium assayed at the indicated times after infection. At days 3 and 4, HMGB1 concentration was so high that the hcDNA probe was saturated, requiring a tenfold dilution of the sample for determination of HMGB1 concentrations (right panel). (B) Gel and calibration curve, obtained with known amounts of pure HMGB1 under identical conditions.

The situation is slightly different when assaying HMGB1 in culture medium or biological fluids, which contain other proteins that can interact with DNA. Such proteins result in a non-specific background that can be very high and obscure any specific signal. However this background can be decreased or even suppressed by addition of a large excess of a non-radioactive competitor DNA thanks to the specificity of the interactions between hcDNA and HMGB1 (or HMGB2), with a binding constant at least three orders of magnitude higher than that of any other protein tested (including hcDNA-specific proteins such as single HMG-box domains, Sry, or p53 [22]). When present, the non-specific and the lower-specificity proteins bind to the non-radioactive DNA in excess, while HMGB1 binds specifically to the labeled hcDNA. Such conditions were used here to detect HMGB1 in the culture medium of human epithelial cells infected by Herpes Simplex Virus type 2 (HSV-2) (Figure 2). The basal level of HMGB1 detected in non-infected cells is due to the necrosis background of the cultured cells, while its increase in infected cells reflects the release of HMGB1 secondary to HSV2-induced necrosis (C.B., manuscript in preparation). In this experiment the addition of 100 ng non-radioactive competitor double strand DNA plus 10 ng single strand, i.e. a competitor/hcDNA ratio of about 1000, was sufficient to eliminate the non-specific noise mostly due to the presence of fetal calf serum in the medium, allowing us to detect HMGB1 at a concentration of 1 pg/µL.

**Figure 3:**
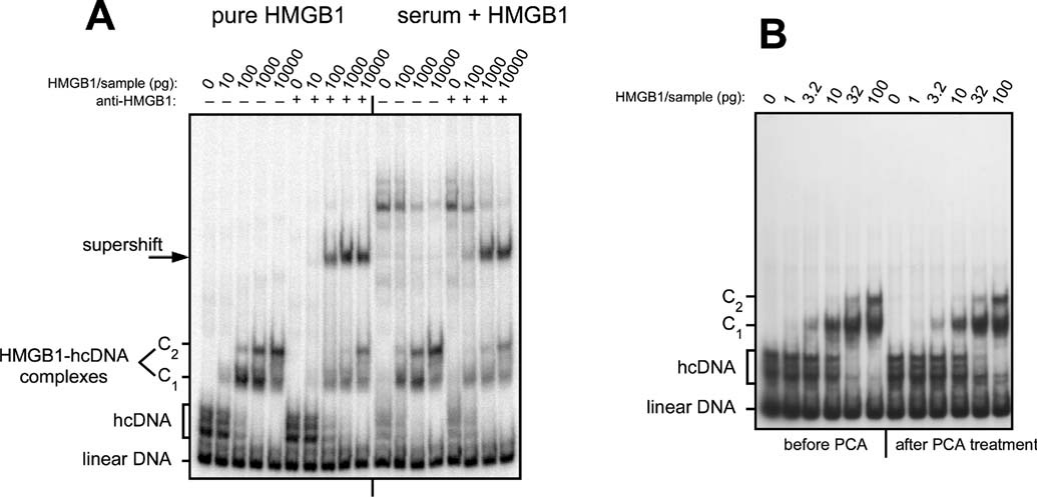
Detection of HMGB1 in human serum. (A) On the left half of the gel, complexes obtained with increasing amounts of pure HMGB1: 0 pg, 10 pg, 100 pg, 1 ng, 10 ng (competitor DNA: 2 µg double strand plus 1 µg single strand per sample). The next five samples contain the same amounts of HMGB1 plus an anti-HMGB1 antibody, showing the supershift of the specific complexes (since pure HMGB1 was used here, and as antibodies were polyclonal and present in excess, the partial supershifting is most likely due to a low or moderate affinity of part of the antibodies). On the right-hand side of the gel, the same experiment was performed with the same amounts of HMGB1 and competitor DNA mixed with 1 µL of serum from a healthy individual. The overall pattern of shifted and supershifted bands is identical, over a background that contains a number of non-specific extra bands, despite the use of a large amount of nonspecific competitor DNA. (B) Purification of HMGB1 by PCA treatment. The left-hand part of the gel shows the interactions of hcDNA with the indicated amounts of pure HMGB1, from 1 pg to 100 pg. On the right-hand part of the gel, samples containing the same concentrations of HMGB1, plus 50 mg/mL BSA to mimic an HMGB1-free serum, were treated with PCA and neutralized as described in Materials and Methods, then assayed for hcDNA binding. Note that the hcDNA-binding activity is recovered without any loss after the PCA treatment procedure.

In practice, when samples to be tested contain high concentrations of various proteins, the nonspecific background becomes more difficult to decrease. This is especially true for human serum, which contains an extremely high protein concentration, about 70 mg/mL. In that case the detection of hcDNA-HMGB1 complexes formed with 1 µL of serum requires the addition of very large amounts of competitor DNA, 2 µg double-strand plus 1 µg single-strand per sample, such amounts of competitor being absolutely required to avoid smearing. Despite the fact that the low-affinity binding of HMGB1 to single-strand DNA results in a decrease of the signal (as observed when comparing the equivalent calibration samples in Figs. 1, 2, 3), under such conditions it is possible to detect 10 pg of HMGB1 per sample (Figure 3A). As can also be seen (Fig. 3A), several other serum proteins bind to hcDNA and result in a significant background and a decrease of the amount of probe available to form complexes with HMGB1 (this appears to be due to the high amount of serum proteins able to bind single-stranded DNA, combined to the fact that the DNA double helix is partially unwound in hcDNA with unpaired bases accessible at the level of the hemicatenane [30]). In short, optimization of the signal-to-noise ratio was found to require a step to purify HMGB1 from other serum proteins. An often-used possibility was to purify HMGB1 by chromatography on ion-exchange columns (heparin is frequently used for that purpose [11]), or by removal of high molecular weight proteins by filtration on membrane (e.g. Centricon) [5]. Instead we took advantage of the property of HMGB1 to be soluble under acidic conditions, whereas most proteins precipitate in acid [31]. This striking and quite unusual property is shared by all proteins of the “High Mobility Group” by definition, as they were first discovered and purified based on their solubility in acid [1]. Most serum proteins instantly form a white precipitate when perchloric acid is added to serum to a final concentration of 3%, while HMGB1 remains in solution. After centrifugation the supernatant is recovered, neutralized with sodium hydroxide, and HMGB1 assayed by band-shift. Figure 3B shows that identical amounts of HMGB1-hcDNA complexes are detected before and after PCA treatment, showing that HMGB1 has quantitatively remained in the supernatant, with its hcDNA-binding activity fully preserved and recovered after treatment with perchloric acid, despite the fact that this is one of the strongest acids known.

**Figure 4:**
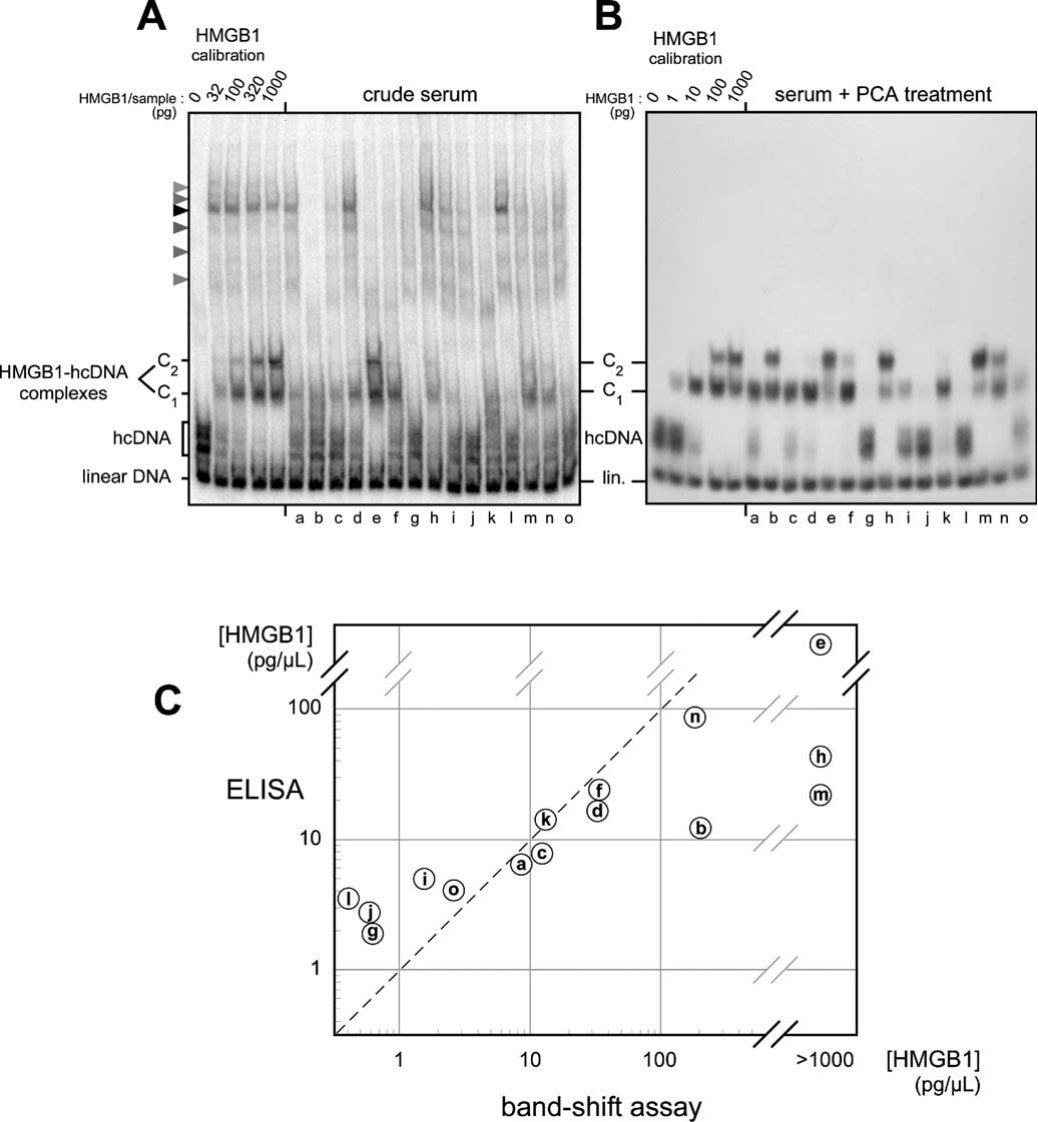
HMGB1 assay in serums from patients. (A) without PCA treatment, or (B) after sample purification by PCA treatment. On each gel the five samples on the left, prepared with known amounts of pure HMGB1, were used to calibrate the method. The 15 serums tested are labeled a-o, serum a originating from a healthy individual, serums b-o from randomly selected patients in intensive care unit (d,g,h,i,k,l,n: sepsis; b,c,e,f,j,m,o: septic shock). Note the lower sensitivity, higher background, and non-specific bands (arrowheads) obtained with crude serums, as compared with the strong increase in sensitivity and marked background decrease after treatment of serums with PCA. (C) Comparison of band-shift assay with ELISA. Concentrations measured by ELISA are plotted against band-shift assay data obtained after PCA treatment of serums, on a double logarithmic scale. Each circled letter on the diagram represents the corresponding serum as assayed in (B).

The potential of the technique is illustrated in Figure 4 with serums from patients hospitalized for acute respiratory distress syndrome with sepsis in the intensive care unit of Saint-Antoine Hospital in Paris. Two series of tests were made in parallel, the first one by assaying the serums directly, the second by pretreatment of the same serums with PCA as described above. Two conclusions can be drawn: first, with unfractionated serums the technique is already extremely sensitive as it allows one to detect 10 pg HMGB1 in a very limited amount of sample (1 µl); second, PCA pretreatment of the serums generally gives qualitatively similar results but leads to a striking reduction of the background and to a tenfold increase in sensitivity, with a detection limit of less than 1 pg HMGB1 per microliter of serum. Despite the small number of serums tested here, one can already observe the wide variations of HMGB1 concentration from one patient to another, from undetectable levels (patients g, j, l) up to more than 1000 pg/µL (patients e, h, m). One can also note the presence of HMGB1 at 10 pg/µL in the control serum from a healthy individual (serum a), as was already observed by another method [15].

In parallel, HMGB1 was assayed using a commercial HMGB1-ELISA kit to compare the two techniques. Result are shown in Figure 4C, circled points a - o representing the results obtained for the same 15 serums, plotted on a double logarithmic scale with band-shift data in abscissa and ELISA in ordinate. Globally the data obtained by both techniques are in very good agreement, most of the points lying near the diagonal. Exceptions include low-concentration samples, lower than 2 pg/µL, for which the ELISA gives higher values than the band-shift assay. This is very likely due to the ELISA background that shows up at low HMGB1 concentrations. Other exceptions are found at high HMGB1 concentration. Samples e, h, m contain more than 1000 pg/µL HMGB1 as judged by band-shift assay (Fig. 4B), but ELISA fails to detect the high concentration in h and m. Similarly sample b also gives a much lower value by ELISA than by band-shift assay. Interestingly the three samples, b, h, m, also gave lower results by band-shift when crude serums were assayed without PCA treatment (Fig. 4A). This strongly suggests the presence of factor(s) that interact with HMGB1 and inhibit its binding both to hcDNA and to antibodies, but are dissociated and most likely precipitated by PCA. This observation is very similar to those made previously by Urbonaviciute et al. [15] who showed that serums can contain factors that mask HMGB1 and interfere strongly with ELISA.

## Discussion

The new method of detection of HMGB1, based on its interactions with hemicatenated DNA loops (hcDNA) as analyzed by band-shift assay, is particularly sensitive. In complex media such as serum, after selective removal of most proteins other than HMGB1 by PCA precipitation, HMGB1 concentrations of 1 pg/µL or lower can be accurately measured. With specimen containing low concentrations of proteins, for example with cerebrospinal fluid or bronchoalveolar lavage (data not shown) or cell-culture medium, the same sensitivity is obtained without the need for PCA precipitation.

This method presents several advantages over other techniques. First, it requires only one microliter of sample per assay, or a few microliters when the PCA precipitation protocol is used, which can be particularly useful with samples other than serum/plasma that may be available in very limited volumes only. Second, the background of the ELISA technique makes it difficult to accurately measure HMGB1 concentrations below 5 pg/µL, whereas a concentration of 1 pg/µL gives a clear specific signal for HMGB1 binding to hcDNA as shown in Figure 4B. Third, several protein factors present in serum or plasma, including anti-HMGB1 antibodies, have been shown to bind HMGB1 and interfere with ELISA [15]; this is observed again with some of the samples tested here, especially those with very high HMGB1 concentrations, most of which are strongly under-estimated by ELISA. With such samples the acidic PCA treatment, which dissociates HMGB1-protein complexes and most likely precipitates the interfering factors, proves very useful to measure the total HMGB1 concentration. Therefore we wondered whether the PCA treatment might also be used to prepare samples for ELISA. However preliminary data (not shown) showed that this treatment results in a decrease by up to 50% of the ELISA signal. This decrease is not due to HMGB1 loss during PCA treatment since no signal decrease is observed for hcDNA binding (Fig. 3B). Neither is it due to an interference of the perchlorate ions with the ELISA, as dilution of the samples did not lower the effect (not shown). Therefore it seems likely that a change in HMGB1 properties occurs upon acidic treatment, as previously observed in other cases [32]–[34], and results in a decrease of its affinity for the antibodies used in the ELISA.

The assay is easy to perform once the radioactive hcDNA probe is ready for use. A single person with only basic electrophoresis equipment can assay 20 samples per gel. In addition to its simplicity, a further advantage of gel electrophoresis, whether performing band-shift assays like here or SDS gels for western blots, is that it provides a direct visual control of the background and of the specificity of the signal. The preparation of hemicatenanes is the only step of the technique that is not of common practice. It has now been described in great detail, however, and should pose no particular difficulties to molecular biology laboratories. The whole process, DNA fragment labelling with ^32^P, hcDNA formation, purification by gel electrophoresis, and electroelution, can be performed in a day. The labeled hcDNA preparation can then be used for a full month, with only a 4-fold decrease in specific activity, as each assay only requires a very small amount of probe (∼5000 cpm, i.e. ∼100 pg). Using ^32^P to label DNA is a well-established, easy to monitor, and convenient way to obtain probes of very high specific activity, allowing the detection of minute amounts of material, with the further advantage that the amount of radiolabeled material in the bands can quickly and easily be quantified by autoradiography or with a phosphorimager.

In addition to HMGB1, several other proteins have been found to bind hcDNA with high affinity. The two other HMGB proteins, HMGB2 and HMGB3, are extremely similar to HMGB1 both in terms of sequence and of biochemical properties. HMGB2 has hcDNA-binding characteristics that are practically undistinguishable from those of HMGB1 [21], [26], and given the sequence similarity this is most probably also the case for HMGB3. Whereas HMGB1 is ubiquitous, HMGB2 and HMGB3 are expressed in specific organs. In the mouse HMGB2 is primarily found in the thymus and testes [35], and HMGB3 in bone marrow [36]. HMGB1 is expressed to very high levels and is one of the most abundant non-histone proteins in the nucleus. In contrast HMGB2 and HMGB3 are much less abundant than HMGB1 and have not been demonstrated to be significantly present in serum (see e.g. western blots in [5]). In any case, HMGB1, HMGB2 and HMGB3 probably share the same extracellular activity since, in addition to their similarity, the 20 amino-acids domain of HMGB1 that was shown to be the determinant of its cytokine activity [37] is 95% conserved in HMGB2, and 90% in HMGB3.

Other proteins that bind hcDNA with high affinity include single HMG-box domains and proteins containing the HMG-box domain of homology such as the sex-determination factor Sry, tumor suppressor p53, as well as the hcDNA-binding proteins present in the serum as shown in the present work. However, such non-HMGB1/2/3 proteins would not be detected by the assay for several reasons. First, our previous studies have shown that the affinity of HMGB1 for hcDNA is at least three orders of magnitude higher than the affinity of single HMG-box domains, Sry [22], or p53 [38], and that these proteins would be displaced by the large amount of nonradioactive competitor DNA present in the assay, as only HMGB1/2/3 remain bound to hcDNA under the high concentrations of competitor DNA used [22]. Second, as mentioned above HMGB1 is extremely abundant, whereas p53 or proteins containing an HMG-box domain are rare. Third, being soluble in acid is quite exceptional for a protein [31], therefore most if not all extra proteins would be precipitated by PCA as is visible in Fig. 4A,B for hcDNA-binding proteins from serum.

As HMGB1 is found methylated, phosphorylated, or acetylated in some specific cases, one may ask whether modified forms would be detected with the same yield as the unmodified form. This question can be asked for all types of assays and clearly deserves further investigation. However reports on the effects of acetylation [39], [40] or methylation [41] failed to show dramatic changes in DNA binding properties of modified HMGB1. Furthermore we have shown that mutations of the intercalating residues of HMGB1, i.e. the amino-acid residues that have been shown to be important for specific binding of distorted DNA structures, have little effect on binding to hcDNA [22]. Therefore it is likely that post-translational modifications of HMGB1 will not dramatically interfere with its affinity for hcDNA.

We only assayed here a very small series of serums, as a test and an example to illustrate the potentialities of the technique. A medically-oriented study using this technique on a large series of patients with well-defined diseases is now in progress in our laboratory. For the moment we can only point out the presence of HMGB1 at a significant concentration (10 ng/mL) in the serum of a healthy control, and the undetectable levels of HMGB1 in the serums of some patients with sepsis. These observations are in good agreement with those recently reported by Angus et al. [42], which suggested that the diagnostic value of assaying HMGB1 concentrations in plasma from patients needed to be studied more deeply [43].

The technique could be further used to study the binding of molecules or macromolecules to HMGB1, as it has been strongly suggested that the extracellular functions of HMGB1 often depend on its interactions with other molecules, such as bacterial lipopolysaccharides, phosphatidylserine, CpG DNA, or cytokines ([44]–[48]; Sylvain Thierry, personal communication). Provided that their interactions do not interfere with hcDNA binding, the band-shift assay described here might again constitute a very sensitive technique to detect HMGB1 partners and to study their interactions.

This technique is among the first practical uses of a DNA nanostructure. DNA nanotechnology has been a very active and promising domain of research, as the properties of DNA are particularly suited to the production of various structures such as nanoobjects, DNA arrays, or nanomechanical devices [49]. Similarly, the development of nucleic acid species engineered to bind various molecular targets is a very active field. These DNA aptamer structures are usually obtained through several rounds of selection for the highest possible binding affinities, as in the SELEX procedure, which confers them very promising therapeutic properties [50]. While initially not a product of SELEX, hcDNA is a DNA structure very similar to an aptamer that will help understand the functions of HMGB1. Furthermore, like for aptamers, the possible therapeutic effect of hcDNA in pathologies involving HMGB1 will also deserve investigation.
